# Supplementary material for: Are carbon emissions trading and green financial instruments synergistic? -Comprehensive quantitative research based on content analysis
Source: PLoS One. 2024 Mar 7;19(3):e0298601. doi: 10.1371/journal.pone.0298601 (PMC10919601; doi:10.1371/journal.pone.0298601)
Supplement: S1 Data — (ZIP) [file pone.0298601.s001.zip › Data/地级市Z_F工作报告.zip!/┴╔─■/╜⌡╓▌╩╨/└┤╫╘╣1⁄2╓┌║┼í╛┬φ┐╦╩2╛▌═°í┐_2019.docx]

各位代表：

　　现在，我代表市政府向大会报告工作，请予审议，并请市政协各位委员提出意见。

　　一、2018年工作回顾

　　过去的一年，在省委、省政府和市委的坚强领导下，在市人大及其常委会和市政协的监督支持下，全市上下深入贯彻落实习近平新时代中国特色社会主义思想和党的十九大精神，深入贯彻落实习近平总书记在辽宁考察时和在深入推进东北振兴座谈会上的重要讲话精神，坚持稳中求进工作总基调，坚定不移践行新发展理念，重实干，强执行，抓落实，全市经济平稳运行，发展质量持续提升，社会事业不断进步，人民生活日益改善。

　　初步核算，全市实现地区生产总值1192.4亿元，增长6.2%；一般公共预算收入100.3亿元，增长9.9%；固定资产投资下降5.4%；规模以上工业增加值增长2.4%；社会消费品零售总额631.4亿元，增长4.8%。城镇和农村常住居民人均可支配收入分别达到32490元和15384元，分别增长6.8%和6.1%。居民消费价格涨幅控制在2.2%以内。

　　（一）坚持深化改革，内生动力持续增强

　　供给侧结构性改革深入推进。关闭30万吨以下煤矿2座，去产能19万吨。完成煤改电项目25个。商品房去化周期9.7个月。新增电力市场化交易准入企业59户。新增“个转企”725户，培育“小升规”企业32户。全市减免各项税收33亿元。政府债务化解取得重大阶段性成果，严厉打击非法集资和金融、医保诈骗，重大风险隐患总体可控。

　　深化“放管服”改革。推进政务服务“一网一门一次”，网上可办率达到72%，91个窗口单位实行双休日“预约服务”。推进工商登记全程电子化，实行“32证合一”“证照分离”，全市新增市场主体3.6万户，总数达到17.9万户。

　　营商环境不断优化。实施“五个一工程”，647项政务服务事项执行一套标准，清理各类证明事项125项，为投资5000万元以上项目配备“项目管家”，8890政务便民服务平台在全省率先投入使用。公开发布行政审批中介服务事项清单，全面清理规范各类中介服务。

　　关键领域改革扎实推进。完成机关和事业单位机构改革任务，政府机构由原来的37个部门优化整合为33个，事业单位由原来的352家精简整合至35家，18家经营性事业单位转企改制，新组建锦勤、担保、城建3个国有企业集团。完成12户央（省）企“三供一业”管理权移交。推进厂办大集体改革。深化县乡财政管理体制改革，全市乡镇全部建立金库并稳定运行。实行“三医联动”改革，公立医院综合改革成果得到国务院通报表扬。推动农村集体产权制度改革，96%的行政村完成集体资产清产核资，农村承包地确权登记颁证工作全面展开。

　　（二）坚持创新驱动，发展活力不断释放

　　科技创新能力持续提升。研发经费支出占地区生产总值比重达到1.15%，科技进步对经济增长的贡献率达到54.5%。新增高新技术企业30家，总数达到121家。科技成果转化62项。成功举办首届锦州市创新创业大赛。七里河经济开发区获批省级高新区，北镇农业科技园区被认定为省级园区，凌海市获评全国农村创业创新典型县。

　　人才引进培育力度加大。出台我市“2+21”人才政策。实行柔性引进人才，一批专家学者、高端人才助力锦州发展，聘请12位院士为锦州产业发展顾问和项目建设顾问，全市院士工作站达到5个。培育9个高层次科技创新创业团队，17名优秀人才、4个优秀团队入选省“兴辽人才计划”，6人获评省级优秀专家。

　　国家级高新区创新引领作用增强。中科院高品质稀土材料制备及应用等10个项目签约入驻大学科技园。华为辽宁大区（锦州）云计算中心项目进展顺利。奥鸿药业获评“2018中国化学制药行业工业企业综合实力百强”。

　　（三）坚持统筹协调，发展质量稳步提升

　　统筹区域经济协调发展。抢抓全省“五大区域发展战略”机遇，全面实施沿海经济带、县域经济和城区经济三年攻坚计划。狠抓沿海经济发展。临港产业不断壮大，汉拿电机汽车发电机及起动机项目被工信部评为智能制造试点示范项目，东方雨虹、阳光能源被确定为全国第三批绿色制造企业。狠抓县域经济发展。扎实推进乡村振兴战略，组织实施5个方面138项攻坚任务。规模以上农业产业化龙头企业达到88户。温氏集团、河南牧原集团等百万头生猪养殖项目先后落地。狠抓城区经济发展。太和区神工、天工半导体项目纳入国家半导体材料中长期发展规划，古塔区大商锦绣前程、凌河区大润发等商业项目开业运营。

　　统筹推动三次产业提质增效。现代农业加快发展。完成种植业结构调整11.2万亩，粮食总产量达到40亿斤。新增节水灌溉10.4万亩，主要农作物综合机械化率达到88%。规模化养殖率达到39.6%。耕地保护、集约节约用地不断加强。及时果断有效处置非洲猪瘟疫情。工业转型升级步伐加快。德瑞克燃料电池等24个项目列入省重大工业项目计划。中石油锦州石化公司25万吨烷基化装置等一批重大项目开工建设。华润锦州电厂主体工程完工，锦阜高、锦承铁路扩能改造进展顺利。表彰选树锦州银行等50户功勋企业，新增省“专精特新”产品（技术）23个、辽宁名牌产品14项。现代服务业发展势头良好。窟窿台批发市场被认定为全国公益性农产品示范市场。同盛居小菜等5家企业获评“辽宁老字号”，沟帮子熏鸡等4个特色产品入选“辽宁礼物”。北镇与京东合作建立“京东·中国特产·北镇馆”。全域旅游示范区创建工作稳步推进，完成辽沈战役纪念馆园区环境升级改造，成功举办冰雪温泉节、古玩节等旅游节庆活动。渤海银行、华泰人寿、中银保险在锦设立分支机构，大地保险区域中心落户我市，凌博信息在辽股交成功挂牌。

　　（四）坚持开放合作，开放型经济稳中向好

　　对外开放合作不断扩大。出台《锦州市关于深度融入共建“一带一路”推动形成全面开放新格局的实施意见》，构建开放发展政策体系。加强与苏州对口合作，建立常态联络机制。密切与省内沿海等兄弟城市及内蒙古赤峰市交流合作。中欧海铁班列实现常态化，国际贸易“单一窗口”全面运行。锦州港完成货物吞吐量1.08亿吨，集装箱运输150万标箱。锦州湾机场新开通锦州—武汉—深圳、锦州—烟台—成都2条航线。全市进出口总额225.7亿元，增长36.4%。光伏、汽车零部件产业基地获评省级外贸转型升级基地。

　　招商引资项目建设年活动成效明显。积极走出去、请进来，面向长三角、珠三角、京津冀等重点地区和日韩俄等国家开展专题招商行动，举办“闽商锦州行”等系列大型招商活动。全年实际到位内资190亿元，实际利用外资5800万美元。阳光能源1GW太阳能电池组件一期投产。中海油LPG物流项目签约落地，海尔物流、威涛物流等项目加快建设。碧桂园、恒大等知名企业入驻我市。

　　（五）坚持绿色发展，城乡面貌日益改善

　　推进“三河共治、三山共建、两环一带建设”。南山公园二期工程竣工，中环西路小凌河大桥、滨河路四期等重点工程开工建设，“蓝色海湾”修复工程扎实推进，龙海广场竣工开放。

　　城乡人居环境持续改善。启动新一轮城市总体规划修编。朝凌客专锦州段建设进展顺利。锦州汽车客运南站、滨海新区客运中心即将投入使用。新建改造城市道路及小街小巷82条，农村公路617公里，各类公厕324座。黑山县被命名为“四好农村路”全国示范县。智慧化城市监管平台投入运行，城市精细化管理水平不断提高。全市人工造林8.8万亩，封山育林4万亩，治理水土流失3.2万亩。

　　污染防治强力推进。狠抓中央生态环保督察及“回头看”反馈意见整改落实，有效解决一批突出环境问题。完成新建、提标改造白沙湾、义县等污水处理厂5座，治理常屯河等黑臭水体4条，实施五里明渠改造工程。完成国家海洋督察反馈整改任务。全面推行河长制。拆除10吨以下燃煤小锅炉202台，新增新能源公交车66台，北镇暖春生物质发电项目竣工。实施秸秆禁烧。空气质量优良率达到74%。开展农村环境集中整治，创建省级生态乡镇1个、生态村10个。

　　（六）坚持共享发展，群众获得感明显增强

　　全市一盘棋，全民总动员，全力推进全国文明城、国家卫生城、国家食品安全示范城“三城联创”。深入开展系列主题活动，着力解决公共服务、公共环境、公共秩序、社会风气等方面的突出问题，市容市貌明显改观，交通秩序不断好转，市民文明素质显著提升，涌现出“最美大学生”丁慧等新时代优秀典型，创城工作取得实实在在的成效。

　　脱贫攻坚扎实推进。严格坚持“六个精准”，全力推进“五个一批”，实施产业扶贫项目172个，投入扶贫专项资金9466万元，发放扶贫贷款3.1亿元，实现9800人脱贫、59个贫困村销号。建档立卡贫困家庭未就业高校毕业生全部就业，贫困人口大病后续医疗保障进一步加强。

　　社会保障水平稳步提高。新增就业1.7万人，城镇登记失业率4.31%。养老金按时足额发放，特殊群体各项基本保障标准全面提高。养老三年行动计划启动实施。贫困残疾人家庭无障碍改造1021户。建成农村饮水安全工程41处。棚户区改造开工4925套，完成农村危房改造3691户。

　　教育事业不断发展。开展学前教育建设年活动，普惠性幼儿园学位覆盖率超过70%。实现义务教育发展基本均衡县（市、区）全覆盖。普通高中质量提升工程成效明显。职业教育长足发展，产教融合、校企合作不断深化。支持驻锦高校建设，产学研一体化有效推进。

　　公共卫生服务持续强化。扎实开展基层卫生建设年活动，百名医生下派支援乡镇卫生院。推进紧密型医联体建设。公立医院药占比控制在30%以下。

　　开展文化建设年活动。隆重举行纪念改革开放40周年、纪念辽沈战役胜利暨锦州解放70周年系列活动，文化惠民演出精彩纷呈，文化产业蓬勃发展。市文化艺术中心主体完工。市群众艺术馆荣获“全国非物质文化遗产保护先进单位”称号。成功举办锦港杯国际帆船邀请赛、全国少年田径锦标赛和北镇国际摄影大展等活动，提升了锦州的知名度和美誉度。

　　平安锦州建设扎实推进。深入开展扫黑除恶专项斗争，打掉各类黑恶势力团伙38个。开展“数据警务、智慧警务”和“雪亮工程”建设，实施派出所基础建设工程，打防能力显著增强。全市刑事案件同比下降10.4%，万人发案率全省最低，群众安全感满意度进一步提高。人民调解工作不断深化，深入实施信访矛盾减存控增三年攻坚计划，解决群众办房照难等一大批信访问题。狠抓安全生产责任落实和专项治理，依法稳妥处置各类矛盾隐患，社会大局总体稳定。

　　(七)坚持务实担当，政府自身建设全面加强

　　把政治建设放在首位。始终高举习近平新时代中国特色社会主义思想伟大旗帜，树牢“四个意识”，坚定“四个自信”，坚决做到“两个维护”，在思想上政治上行动上同以习近平同志为核心的党中央保持高度一致。扎实抓好中央巡视反馈意见整改落实，强化“一岗双责”，推进全面从严治党向纵深发展，坚决贯彻落实中央重大决策，坚决贯彻落实省委、省政府和市委部署要求。

　　自觉接受市人大及其常委会的法律监督、工作监督和市政协的民主监督。认真听取各民主党派、工商联、无党派人士和人民团体的意见。146件人大代表建议、233件政协提案全部按时办结。关于整治架空通讯线路提升城市品位的人大议案得到有效落实。

　　始终把纪律规矩挺在前面。认真落实中央八项规定精神及省、市实施细则，切实解决群众身边的腐败问题。推进依法行政，规范行政行为，完善“三重一大”决策机制，加大跟踪审计力度，严格督查问责和绩效考评。强力推进“重强抓”专项行动，对标对表省政府任务指标，推动压力层层传导、责任层层落实。

　　国家安全、人防、气象、地震和民兵预备役等工作不断加强，双拥共建、民族宗教、外事侨务、慈善、档案、供销、地方志和住房公积金管理等工作取得新成果。

　　各位代表，成绩来之不易，这是省委、省政府和市委正确领导的结果，是市人大、市政协监督支持的结果，是全市人民团结奋斗的结果。在此，我代表市政府向全市人民，向人大代表、政协委员，向各民主党派、工商联、无党派人士和各人民团体，向驻锦中省直单位、人民解放军和武警部队，向所有关心支持参与锦州振兴发展的朋友们，表示崇高的敬意和衷心的感谢！

　　回顾过去一年的工作，我们清醒地认识到，发展不充分不平衡的问题尚未解决。经济总量小，项目少。新兴产业占比偏低，产业链延伸不够。区域发展不协调，先导区辐射带动作用不强，县域经济短板突出，城区经济活力不足。一些领域体制机制深层次问题尚未破解，营商环境仍存在薄弱环节。三大攻坚战任务艰巨，民生领域历史遗留问题较多。特别是思想解放不够，开拓创新意识不强，不担当、不作为等现象不同程度存在。针对这些问题，我们一定要以对党和人民高度负责的态度，勇于革新，敢于斗争，善于作为，竭尽全力把锦州的事情办好，不负人民的重托！

　　二、2019年主要工作安排

　　2019年是新中国成立70周年，是决胜全面建成小康社会的关键之年，也是锦州全面振兴、全方位振兴的开局之年，做好今年各项工作意义重大、影响深远。

　　今年政府工作的总体要求是：高举习近平新时代中国特色社会主义思想伟大旗帜，全面贯彻党的十九大和十九届二中、三中全会精神，深入落实习近平总书记在辽宁考察时和在深入推进东北振兴座谈会上的重要讲话精神，认真落实中央和省、市委经济工作会议各项部署，统筹推进“五位一体”总体布局，协调推进“四个全面”战略布局，坚持稳中求进工作总基调，坚持新发展理念，坚持推进高质量发展，坚持以供给侧结构性改革为主线，聚焦“六项重点工作”，加快补齐“四个短板”，继续打好三大攻坚战，深入实施“五大战略”，全力打造实力、法治、文明、生态、幸福锦州，解放思想、开拓创新，科学施策、扎实工作，以新气象新担当新作为推动锦州加快实现全面振兴、全方位振兴。

　　今年全市经济社会发展的主要预期目标是：地区生产总值增长6.6%，一般公共预算收入增长7%，固定资产投资增长8%，规模以上工业增加值增长5%，社会消费品零售总额增长5%，城乡居民收入和经济增长同步，居民消费价格涨幅控制在3.5%以内。

　　在实际工作中，我们将牢牢抓住项目建设这个核心，以飞地经济为载体，以“重强抓”专项行动为手段，以营造一流营商环境为保障，统筹推进三大经济区域协调发展，进一步稳就业、稳金融、稳外贸、稳外资、稳投资、稳预期，确保主要经济指标增速不低于沿海六市平均水平，力争部分指标增幅位居全省前列，保持经济持续健康发展和社会大局稳定。

　　今年，要重点做好以下七个方面工作：

　　（一）狠抓深化改革，增添全面振兴新活力

　　持续优化营商环境，围绕供给侧结构性改革，完善改革思路，做实改革举措，全面深化各领域改革。

　　以机构改革“三定”方案为基础，调整政府部门权责清单，完善工作流程和岗位职责。继续深化“放管服”改革，全面推进“多证合一”“证照分离”和政务服务“一网一门一次”改革，强化“双随机、一公开”事中事后监管。工程建设项目审批时限压缩至90个工作日，一般性企业开办时间缩短到3.5天。市县两级政务服务事项按照“应进必进”原则全部进入综合性政务服务大厅，网上可办率不低于80%，公共服务事项开通150项，100个高频事项实现“最多跑一次”。持续开展“办事难”专项整治和“帮扶企业”专项行动，全面推行配备“项目管家”，畅通政企沟通渠道，构建亲清政商关系，营造公平正义的法治环境、高效透明的政务环境、竞争有序的市场环境。

　　深入推进供给侧结构性改革。依法依规加强房地产市场监管，整饬房地产乱象，规范房地产业健康发展。增加保障性住房供给，满足不同群体住房需求。落实减税降费政策，切实减轻企业负担。

　　深化国资国企改革。加强组织领导，推进政企分开、政资分开，加快实现从管企业向管资本转变。规范法人治理结构，建立和完善现代企业制度，解决人员能进不能出、待遇能高不能低、职务能上不能下的问题。积极推进国有企业集团混合所有制改革。39户厂办大集体改革全部完成。

　　做大做强民营经济。深入贯彻习近平总书记在民营企业座谈会上的重要讲话精神，全面落实我市扶持民营经济发展31项新政，着力解决融资难融资贵、人才匮乏、办事难等问题。依法保护民营企业和企业家合法权益，大力弘扬企业家精神，营造尊重、激励和保护企业家干事创业良好社会氛围。培养壮大一批民营企业，持续激发市场主体活力，全年实现“个转企”800户、“小升规”50户。

　　坚持把解决“三农”问题作为工作的重中之重，全面深化农村改革，切实落实强农、惠农、富农各项政策。盘活“四荒地”等村集体资产，壮大集体经济。落实集体土地“三权分置”办法，千方百计增加农民收入。深化县乡财政管理体制改革，不断提升乡镇财政保障能力。

　　开发区、园区承载着锦州振兴发展的希望。要全面落实开发区、园区体制机制改革各项政策，进一步明晰发展定位和主导产业，盘活闲置厂房场地，优化人员结构，推进扁平化管理，实行档案绩效工资，下放审批权力，建立完善财政管理制度，实行财政单独核算。推进园区整合，减少数量，提高质量，实施“一区多园”，通过“园区+公司”的办法，利用市场化手段，完善园区基础设施，加快建设一批承载能力大、聚集功能强的特色产业园区。

　　（二）狠抓创新驱动，集聚全面振兴新动能

　　坚持把创新作为引领全面振兴的第一动力，依靠创新培育新动能、改造提升传统动能，加快推动实体经济、科技创新、人力资源协同发展。

　　培育壮大八大主导产业。坚持项目园区化、产业集群化，加快石油化工、铁合金、农副产品深加工等传统产业转型升级，大力发展新材料、大数据、生物制药、光伏、汽车及零部件等战略新兴产业，同步推进海洋产业、现代农业和现代服务业加快发展。充分发挥“8+3”产业推进组作用，切实在要素配置、项目建设、“双招双引”、技术创新等方面实现新突破。进一步推进信息化与工业化深度融合。继续实施质量品牌建设工程，着力培育一批工业名牌产品，抓好智能制造、智能服务示范项目建设。积极推进军民融合深度发展。

　　全面提升科技创新能力。增加研发经费投入，科技进步对经济增长的贡献率达到55%。实施高新技术企业培育工程，新增高新技术企业25家。打造创新创业平台，推进国家知识产权运营公共服务平台落户我市。深化与中科院、大连理工等“两院十校”及驻锦高校的战略合作，完善技术转移体系，重点推动一批创新成果在大学科技园落地转化。

　　集聚创新创业人才。全面实施“2+21”人才政策，深入落实柔性引进人才办法，重点培育和引进一批科技领军人才、青年科技人才、高水平创新团队和高层次管理人才，大力培育工匠精神。坚持用事业留人、用感情留人、用待遇留人，不拘一格发现人才、使用人才，让各类人才的创新创造活力在锦州振兴发展中竞相迸发。

　　加快智慧城市建设。依托华为云计算中心，推进智慧城市综合指挥中心和政务信息系统建设，实现数据共享。加速公安、医疗、教育、交通、城管等领域信息化应用，全面提升智慧化管理、智能化服务水平。

　　（三）狠抓协调发展，构建全面振兴新格局

　　深入实施辽宁沿海经济带锦州三年攻坚计划，科学统筹、精准施策，加速形成三大经济区域协调发展、三次产业协同发力的良好局面。

　　加快构筑“2+1”沿海经济发展新格局。激发先导区活力，进一步发挥引领带动作用。推进滨海新区中海油LPG物流、锦州医科大学医疗学院等项目建设。推动国家高新区产城融合示范区建设，万得科技广场、华为云计算中心等项目投入运营。加快凌海“转身向海”步伐，实施临港项目建设，推进临港产业发展。

　　以乡村振兴战略为抓手发展壮大县域经济。坚持农业农村优先发展，夯实粮食生产能力和农业基础，突出优质、特色、绿色等调整优化农业结构，推动农村各产业融合发展。加快培育农业发展新动能，支持各类人才返乡下乡创业创新，拓展农村就业空间和增收渠道，巩固发展“三农”持续向好形势，为经济社会持续健康发展提供有力支撑。要立足县情，发挥比较优势，打好特色品牌，拉长产业链条，着力推动农产品深加工，深化“一乡一品、一县一业”。拓展为“三农”服务的空间，强化为“三农”服务的手段，鼓励支持农民各类合作社建设，支持农产品电商平台建设，提升信息化服务水平。

　　以服务业提质增效带动城区经济提档升级。推进传统服务业转型升级和现代服务业加快发展。培育壮大凌河、古塔夜市等一批特色商业街区，做大会展经济，大力发展金融保险、电子商务、现代物流、健康养老、呼叫中心等现代服务业。擦亮“山海福地锦绣之州”城市品牌，整合红色文化、历史文化、自然生态等要素资源，推动乡村旅游、冰雪旅游、温泉旅游，建设自驾游房车营地，抓好全域旅游规划编制，促进旅游业健康发展。优化金融服务，创新金融产品，支持企业上市，鼓励运用新三板和区域股权市场融资。各城区以服务业为重点，实行差异化特色发展，持续增强城区经济实力。

　　（四）狠抓绿色发展，打造全面振兴新优势

　　贯彻绿水青山就是金山银山、冰天雪地也是金山银山的理念。牢牢守住生态保护红线、环境质量底线、资源利用上线，以人与自然和谐为价值取向，以绿色低碳循环为主要原则，以生态文明建设为基本抓手，大力发展绿色经济，加快传统产业智能化、清洁化、绿色化改造，重点抓好义县再生资源产业园、太和静脉产业园等项目建设，推进生产、生活、生态绿色化。

　　统筹山水林田湖草治理。认真开展第三次国土调查和第四次全国经济普查。深入推进三北防护林等重大生态修复工程。大力植树造林，全年人工造林6.2万亩、封山育林1万亩。实施锦凌水库周边及城区绿化工程，全力打造生态屏障。落实河长制，加强域内河流保护。统筹推进城市污水和垃圾处理。深入开展“五矿共治”专项行动，加大非煤矿山综合治理力度。

　　深入推进“三河共治、三山共建、两环一带建设”。中环西路小凌河大桥、滨河路四期等重点工程竣工，加快推进女儿河王宝山段生态综合治理、滨河路五期等重点工程建设。推进“蓝色海湾”整治修复，打造生态良好、环境宜人的黄金海岸，建设宜居宜游宜业的锦绣之州。

　　（五）狠抓开放合作，构筑全面振兴新高地

　　跳出锦州看锦州，以战略眼光、全球视野谋划锦州开放发展，突出项目中心地位，坚定不移地把招商引资作为推动发展一号工程，以全面开放引领全面振兴。

　　抢抓战略机遇。用足用好东北振兴和辽宁沿海经济带系列政策，积极争取国家和省政策、项目、资金支持。主动融入“一带一路”，大力发展临港经济，持续增加中欧海铁班列货运量。加强与蒙东地区交流合作，积极协调推动“锦赤白珠乔铁路”建设，力争早日打通中蒙俄最新最便捷的大通道。支持锦州港参与辽宁港口资源整合。加强国际贸易“单一窗口”推广应用，促进跨境贸易便利化。积极参与辽宁中国—中东欧“16+1”经贸合作示范区建设，深化与苏州对口合作。强化重点进出口企业政策引导和服务，全市外贸出口增长8%。

　　加大招商引资力度。完善和落实招商引资政策。通过以商招商、产业链招商、乡情招商等方式，紧盯京津冀、长三角、珠三角等重点地区，深耕日韩俄，做大港澳台，压实主体责任，完善考核体系。全年新引进3000万元以上项目170个，其中10亿元以上项目20个。引进内资和外资分别增长10%以上。

　　继续实施项目建设年活动。牢固树立“项目为王”意识，一切围绕项目转、一切围绕项目干。坚持重大项目“一个项目、一名领导、一套班子、一抓到底”工作机制，强化项目建设分类调度和要素保障，实行挂图作战，压茬推进。全年开复工亿元以上项目170个，完成投资240亿元。加快推进中石油锦州石化15万吨针状焦、中信钛业6万吨钛白粉扩能改造、辽宁京东管业100万吨球墨铸管等一批重点项目建设。完成城东220千伏输变电工程。对接国家和省投资项目及引导基金，围绕重大基础设施、新旧动能转换、战略新兴产业等包装储备一批重点项目。

　　大力发展飞地经济。把发展飞地经济作为要素集约、资源集成、力量集聚的重要载体，破解乡镇项目建设难题。年内飞地经济园区要覆盖四个县（市），全部乡镇均有飞地项目落地。滨海新区要探索建设跨省飞地园区。

　　（六）狠抓三大攻坚战，筑牢全面振兴新基础

　　提高政治站位，强化责任担当，瞄准重点难点问题和薄弱环节，全力打好三大攻坚战，为决胜全面建成小康社会奠定坚实基础。

　　坚决打好防范化解重大风险攻坚战。严格执行政府债务限额管理，强化隐性债务风险防控，化解存量，严控增量，严禁违规举债。规范实施存量PPP项目。有效防范国有企业债务风险。加大养老保险基金保费征缴和财政补助力度，严格支出管理，确保养老金按时足额发放。加强对重点领域金融风险的排查、预警和化解，开展互联网金融风险专项整治，依法严厉打击非法集资、金融诈骗、医保诈骗等行为。加强对防范重大风险的宣传教育，落实防范化解风险主体责任，提高风险管控能力。

　　坚决打好精准脱贫攻坚战。今年是脱贫攻坚决胜年，要聚焦贫困地区和贫困群体，做到全覆盖、无死角。对标扶贫标准、对表时间节点、对照质量要求，精准到村、到户、到人，在精准脱贫、稳定脱贫、持续脱贫上下功夫，在制定完善扶贫工作后续政策措施上下功夫。确保完成6100人脱贫、25个贫困村销号、义县省级贫困县摘帽的脱贫攻坚扫尾任务，确保2020年贫困地区和贫困群众同全市一道进入全面小康社会，确保小康路上不少一人、不落一户。

　　坚决打好污染防治攻坚战。着力“巩固气、突破水、治理土”，深入推进中央生态环保督察和国家海洋督察整改落实。实施污染防治攻坚战三年专项行动计划，坚决打好蓝天保卫战、柴油货车污染治理、水源地保护、重污染河流治理、城市黑臭水体治理、渤海锦州段综合治理、农业农村污染治理“七大关键性战役”。推进淘汰建成区20吨以下燃煤锅炉，严格实施秸秆禁烧，环境空气质量持续改善。加强水土流失治理和农田水利建设，实施农村饮水安全提升工程。依法加大锦凌水库等水源地保护力度，落实最严格水资源管理制度，出重拳，零容忍，坚决依法打击有毒、有害物质排入河流和偷排、超排等违法行为。开展农村环境综合整治，推进农村生活垃圾处理、改水改厕、绿化美化工程建设，创建省级生态县，打造美丽乡村。

　　（七）狠抓改善民生，共享全面振兴新成果

　　不断满足人民对美好生活的新期盼，坚持从人民群众关心、让人民群众满意的事情做起，以“三城联创”为重要载体，持续优化公共服务，扎实办好民生实事，着力提高人民生活质量，让振兴成果更多更公平惠及全市人民。

　　积极扩大就业。推动创业带动就业，新增就业1.6万人，城镇登记失业率控制在5%以内，零就业家庭保持动态为零。大力开展职业培训和就业援助，多渠道促进高校毕业生就业。

　　强化社会保障。及时调增特殊群体保障标准。实施“六统一”城乡居民医保制度，保证全市城乡居民享有公平统一的医疗保险政策。为全市生活困难残疾人购买意外伤害保险。落实养老三年行动计划，加快构建多元化居家和社区养老服务体系。

　　优先发展教育，打造辽西教育中心。推动学前教育普惠健康发展，破解“入园难、入园贵”问题。严格规范中小学校办学行为，着力解决中小学生课业负担重、“大班额”问题。优化教育布局。推进“订单式”职业教育，培养高素质劳动者和技能型人才。支持驻锦高校建设和发展。

　　推进健康锦州建设，打造辽西医疗中心。落实《“健康中国2030”规划纲要》，持续提高人民健康水平。深化公立医院综合改革，加强医联体建设，完善分级诊疗和家庭医生签约制度，促进医疗资源下沉，基层医疗卫生机构标准化建设达标率90%以上，为群众提供更加优质的医疗服务。支持锦州医科大学及附属医院和驻锦部队医院创新发展，更好服务辽西人民。

　　推动文化繁荣发展，打造辽西文化中心。发挥专业文艺团体优势，尊重文艺创作规律，创作一批文艺精品。健全公共文化服务体系，实施文化惠民工程。市文化艺术中心投入使用。抓好群众文化节、京剧票友节等重点文化品牌建设，丰富群众文化生活，加快发展文化产业。实施文物保护工程。推进体育事业发展，规划建设国家帆船培训基地。

　　加强和创新社会治理。深入开展扫黑除恶专项斗争，加强公安装备保障，强化队伍建设，严厉打击各类违法犯罪，打造平安锦州。做好法律援助工作，健全公共法律服务体系。做好民族宗教工作，促进民族团结、宗教和谐。做好退役军人事务服务工作，深化国防动员与双拥共建，巩固军政军民团结。深入实施信访矛盾减存控增三年攻坚计划，依法及时解决群众合理诉求。加强应急管理体系建设，做好防灾减灾救灾工作，严格落实安全生产责任制，坚决遏制重特大事故发生，全面提高社会治理水平和治理能力。

　　三、努力建设人民满意政府

　　锦州已经开启全面振兴、全方位振兴新征程，新征程要有新气象新担当新作为。我们要以永不懈怠的精神和奔跑起来的姿态，深入推进政府职能转变，全面提升履职水平，切实做到对人民负责、受人民监督、让人民满意。

　　强化政治建设。深入学习贯彻党的十九大精神，坚持用习近平新时代中国特色社会主义思想武装头脑、指导实践、推动工作。牢固树立“四个意识”，坚定“四个自信”，坚决做到“两个维护”，自觉在思想上政治上行动上同以习近平同志为核心的党中央保持高度一致。加强意识形态工作，站稳政治立场，提高政治站位，强化政治担当，保证政令畅通，确保党中央大政方针、决策部署和省、市委的工作要求落实落地。

　　强化依法行政。严格遵守宪法和法律，落实政府工作规则，依照法定权限和法定程序行使权力、履行职责，主动运用法治手段破解工作中的难题。推动水源保护、市容环境管理、文明行为促进、食品安全监管等重点领域立法。认真执行市人大及其常委会的决议决定，坚持重大事项向人大报告、向政协通报制度，自觉接受人大法律监督、工作监督和政协民主监督，广泛听取各方面意见，认真办理建议提案。完善政府法律顾问制度，健全重大决策合法性审查、风险性评估机制，确保依法科学民主决策。推进政务公开，强化审计监督和督查考评。加强诚信建设，作出的部署必须落实到位，作出的承诺必须坚决兑现。

　　做到清正廉明。严格落实管党治党主体责任和“一岗双责”，持之以恒落实中央八项规定精神和省、市实施细则。加强对土地出让、房地产开发、矿产资源利用、项目建设等重点领域的监管，坚决防范权力寻租、暗箱操作等腐败现象发生。坚持无禁区、全覆盖、零容忍，严厉查处扶贫、社会保障等领域群众身边的腐败问题。坚持有腐必反、有贪必肃，巩固反腐败斗争压倒性态势。抓早抓小，强化教育管理和制度完善，构建不敢腐不能腐不想腐的长效机制。做到秉公用权、依法用权、廉洁用权、为民用权，实现干部清正、政府清廉、政治清明。

　　做到真抓实干。锦州全面振兴、全方位振兴不会是轻轻松松、敲锣打鼓就能实现的，前进的道路不会一帆风顺，我们要忠于职守，敢于担当，奋发有为。要深入群众、深入实际，在人民群众中汲取智慧和力量，不说空话，多做实事，直面矛盾和问题，勇于攻坚克难，重实干、强执行、抓落实。坚决整治庸懒散、不作为、慢作为、乱作为等现象。撸起袖子加油干，干出气势，干出激情，干出百舸争流、千帆竞发的生动局面。

　　各位代表，使命重在担当，实干铸就辉煌。让我们高举习近平新时代中国特色社会主义思想伟大旗帜，在省委、省政府和市委的坚强领导下，不忘初心、牢记使命、永远奋斗，推动高质量发展，共同谱写锦州全面振兴、全方位振兴的新篇章，为加快建设辽西区域中心城市、决胜全面建成小康社会而不懈努力，以优异的成绩向中华人民共和国成立70周年献礼！
